# ncRNAs mediated RPS6KA2 inhibits ovarian cancer proliferation via p38/MAPK signaling pathway

**DOI:** 10.3389/fonc.2023.1028301

**Published:** 2023-01-19

**Authors:** Zhiqin Fu, Chao Ding, Wangang Gong, Chao Lu

**Affiliations:** ^1^ The Cancer Hospital of the University of Chinese Academy of Sciences (Zhejiang Cancer Hospital), Institute of Basic Medicine and Cancer (IBMC), Chinese Academy of Sciences, Hangzhou, Zhejiang, China; ^2^ Department of General Surgery, Cancer Center, Division of Gastrointestinal and Pancreatic Surgery, Zhejiang Provincial People’s Hospital, Affiliated People’s Hospital, Hangzhou Medical College, Hangzhou, Zhejiang, China; ^3^ Key Laboratory of Gastroenterology of Zhejiang Province, Hangzhou, Zhejiang, China

**Keywords:** ovarian cancer, RPS6KA2, circular RNA, miRNA, proliferation

## Abstract

**Background:**

Ovarian cancer is the most lethal gynecology malignancy in the world, therefore, research on the molecular biological mechanism of ovarian cancer tumorigenesis and progression has received widespread attention.

**Methods:**

We identified RPS6KA2 as the prognosis-related gene of ovarian cancer from TCGA, GSE26712 and GSE26193 database *via* bioinformatic analysis. qRT-PCR and western blot detected the differential expression of RPS6KA2 in normal ovaries and ovarian cancer tissues. The biological functions of RPS6KA2 were verified by *in vitro* and *in vivo*. GSEA analysis was used to select candidate signaling pathway of RPS6KA2 which was further verified by western blot. The possible binding sites of RPS6KA2 with miRNAs and circRNAs were predicted by bioinformatics analysis, and then a circRNA-miRNA-mRNA interaction network was constructed.

**Results:**

We found the expression of RPS6KA2 was down-regulated in ovarian cancer tissues. Overexpression of RPS6KA2 could suppress cell proliferation, whereas knockdown of RPS6KA2 had the opposite effects on proliferation. GSEA analysis showed that the MARK signaling pathway was closely associated with RPS6KA2. Bioinformatics analysis and dual-luciferase reporter assay showed that RPS6KA2 was regulated with miR-19a-3p, miR-106a-5p and miR-519d-3p. Further analysis showed that circFAM169A was the common ceRNA of miR-19a-3p, miR-106a-5p and miR-519d-3p. Dual-luciferase reporter assay showed the relationship of circFAM169A and miR-106a-5p and miR-519d-3p. After network analysis, one circRNA-miRNA-mRNA axis (circFAM169A/miR-106a-5p, miR-519d-3p/RPS6KA2) was identified.

**Conclusions:**

We demonstrated that circFAM169A/miR-106a-5p, miR-519d-3p mediated low expression of RPS6KA2 could affect the proliferation of ovarian cancer cells *via* p38/MAPK signaling pathway.

## Introduction

Ovarian cancer remains the first cause of death in gynecological cancer and the major cause of death in women (1, 2). Due to the insidious early symptoms, early diagnosis of ovarian cancer is difficult, and 70% patients are already at the advanced stages at the time of diagnosis (3, 4). Chemotherapy, surgery and targeted therapy are the currently main therapeutic strategies for ovarian cancer (5–8). Despite significant advances of clinical techniques, most patients with advanced stages have experienced a cancer recurrence (9–11). Therefore, it is essential to identify novel biomarkers and elucidate mechanisms about the progression of ovarian cancer.

The ribosomal protein S6 kinase (RPS6K) family is involved in numerous pathways, many of which have pivotal roles in the carcinogenic process. RPS6KA2 is the member of RPS6K family (12–15). Different from other RPS6Ks, RPS6KA2 has dual catalytic domains (16). The first domain is homologous to the cyclic AMP kinase family whereas the second to the phosphorylase kinase family. Both domains contribute to growth factor stimulated autophosphorylation of RPS6KA2 (12). Studies have suggested RPS6KA2 is related to the occurrence and development of prostate cancer (17, 18). However, the precise function of each subtype is poorly known, until now, the function role of RPS6KA2 in ovarian cancer remains unknown.

Circular RNAs (circRNAs) is a subtype of noncoding RNAs (ncRNAs) (19, 20), unlike traditional linear RNAs containing 5’ and 3’ terminal ends, circRNAs molecule have a closed-loop circular structure and are unlikely to be degraded by exonuclease (21–23). CircRNAs are related to cell stability, migration, differentiation, metabolism, and autophagy (24–27). Recent studies have found that circRNAs are aberrantly expressed in breast, ovarian, and non-small cell lung cancer, and affect their prognosis (28–30). CircFAM169A is a member of the family of Circular RNAs. Guo et al. showed dysregulated circFAM169A regulates intervertebral disc degeneration by targeting miR-583 and BTRC (31). However, the role of circFAM169A in the development of ovarian cancer remains to be studied.

In this study, we found that RPS6KA2 showed significant low expression in ovarian cancer tissue and that there was a correlation between the low expression of RPS6KA2 and poor prognosis in ovarian cancer patients. Second, overexpression of RPS6KA2 could inhibit cell proliferation *in vitro*, and markedly suppressed tumor growth *in vivo*. Then we demonstrated that RPS6KA2 inhibited proliferation in ovarian cancer *via* p38/MAPK signaling pathway. Finally, we revealed for the first time the biological function of circFAM169A as upstream of RPS6KA2 in ovarian cancer and regulate RPS6KA2 through miR-106a-5p and miR-519d-3p. This finding might provide new clues for the treatment of ovarian cancer.

## Materials and methods

### Bioinformatics analysis

Pearson’s correlation analysis was used to extract prognosis-related genes of ovarian cancer in each dataset (with the | Pearson R|> 0.5 and p < 0.05). The prognosis-related genes screened from TCGA database and two GEO datasets (GSE26712, GSE26193) were intersected to obtain a shared prognosis-related gene (RPS6KA2). Then, the protein expression of RPS6KA2 in primary ovarian cancer (n=426) and normal tissues(n=88) analyzed by TCGA database. Overall survival (OS) and relapse-free survival (RFS) were shown by KM-plot website (http://www.kmplot.com/). The Human Protein Atlas database (https://www.proteinatlas.org/) was used to show a lower expression level of RPS6KA2 expression in tumor tissues and in normal tissues. The pathway analyses were performed with gene set enrichment analysis (GSEA, http://software.broadinstitute.org/gsea/index.isp). miRNA targeting RPS6KA2 was predicted with TargetScan (https://www.targetscan.org/vert_80/). And circRNA was predicted by circBank database (http://www.circbank.cn/index.html).

### Ovarian cancer specimens

All procedures performed in this study involving human participants were consistent with the ethical standards of the Declaration of Helsinki and its later amendments or comparable ethical standards. The ethics committee of Zhejiang Cancer Hospital approved this study (NO. IRB-2021-124). 40 samples of epithelial ovarian cancer and 17 samples of normal ovaries were collected for RNA extraction from Zhejiang Cancer Hospital (2021.06-2022.12). In addition, 50 formalin fixed and paraffin embedded ovarian cancer specimens from the biobank of Institute of Basic Medicine and Cancer were sectioned for RPS6KA2 immunohistochemistry staining.

### RNA extraction and quantitative real-time PCR

Trizol reagent (Invitrogen, USA) was used to isolate the total RNA from tissue specimens or cells. Nucleoplasmic separation assays were conducted using a nuclear/cytosol fractionation kit (Solarbio, China). Reverse transcription was performed using a transcriptase cDNA synthesis kit according to the manufacturer’s instructions. Then, the purity of total RNA was gauged by applying a NanoDrop 2000 Spectrophotometer. qRT-PCR was implemented to detect the expression level of circRNA, miRNA and mRNA. GAPDH represented endogenous reference. Results were detected with method of 2^-ΔΔCT^. Each sample was tested from three independent experiments.

### Immunohistochemistry and H-score

A standard procedure was performed for IHC staining. After de-paraffinization and quenching of endogenous peroxidase, we incubated the sections with 1% bovine serum albumin (BSA) in PBS. Subsequently, the sections were treated with anti-RPS6KA2 antibodies followed by incubation with a goat anti-rabbit-peroxidase conjugated second antibody. DAB (3,3-diaminodbenzidine) substrate was added and the sections were counterstained with hematoxylin. Finally, the mounted sections were observed with a microscope (Leica, Germany). We quantified the data and calculated H-score (H-Score=∑(pi×i)=(percentage of weak intensity×1)+(percentage of moderate intensity×2)+(percentage of strong intensity×3) by Quant Center (v2.1, Hungary) and Halo (v3.0, USA).

### Cell culture

We purchased the human ovarian cancer cell lines IOSE386, HO8910, SKOV3, OVCAR3, A2780 and COC2 from American Type Culture Collection (ATCC, Manassas, VA, USA). All cell lines were cultured in Dulbecco’s modified Eagle’s medium (DMEM) supplemented with 10% fetal bovine serum (FBS) and maintained at 37°C in 5% CO_2_.

### Cell proliferation assays

For Cell Counting Kit 8 (Beyotime, China) assay, cells were seeded into 96-well plates and cultured at 2000 cells per well. The cells were cultured for 24h, 48h, 72h, and 96h respectively, and then, added with 10μg of CCK-8 reagent. After incubation for 2 hours, the optical density (OD) value at 450 nm absorption wavelength of each well was measured in the microplate reader. For colony formation assay, cells were plated into 6-well plates and cultured for 14 days. Finally, colonies were fixed with 4% paraformaldehyde, stained with crystal violet and photographed. An EdU Kit (Beyotime, China) was used to assess the cell proliferation ability. Cells were seeded into 96-well plates and cultured with EdU reagent (1:1000 dilution) for 2 h the next day. Then, 4% paraformaldehyde was used to fix the cells, and fluorescent dye and DAPI were used to stain cells. Cell images were captured and counted by a fluorescence microscope (Nikon, Japan).

### Plasmid transfection

The short hairpin RNA (shRNA) and overexpressing plasmid and their negative control plasmids were purchased from GenePharma company (GenePharma, China). The transfection of ovarian cancer cells (OVCAR3, SKOV3) was performed by using Lipofectamine 2000 (Invitrogen, USA) reagent following the manufacturer’s instructions.

### Western blots

Ovarian cancer cells were harvested and lysed using RIPA lysis buffer (Beyotime, China) with protease inhibitors. The bicinchoninic acid (BCA) protein assay kit was used to quantify the concentration of extracted protein. The Protein samples were electrophoresed on polyacrylamide gels and then transferred to polyvinylidene difluoride (PVDF) membranes (Millipore, USA). The membranes were incubated with primary antibody after blocking with 5% skimmed milk at 4°C overnight. Then, the membrane was incubated with the secondary antibody at room temperature for 1h after rinsing with the Tris-Buffered Salin and Tween buffer solution. The protein bands on the membrane were exposed by the chemiluminescence.

### Nude mouse model of subcutaneous xenograft

Nude mice were purchased and samples were collected in accordance with the guidelines of Zhejiang Cancer Hospital. The 4-week-old female nude mice (five mice/group) were injected subcutaneously with 1×10^7^ ovarian cancer cells. All the mice were sacrificed and tumor lesions were excised and photographed after 4 weeks. The tumor volume (mm^3^) was calculated as length × width^2^/2.

### Dual-luciferase reporter assay

Using pMIR-REPORTTM system (Applied Biosystems, USA) to measure the interactions between mRNA, circRNA and miRNAs. The transcription factor expression plasmid to be detected was co-transfected with the reporter plasmid into the OVCAR3 and CAOV3 cell lines. Dual luciferase activity was tested after 48h using the Dual Luciferase Reporter Assay Kit (Promega, USA), strictly following the manufacturer’s instructions.

### Fluorescence *in situ* hybridization

FISH assay was performed to observe the location of circFAM169A in ovarian cancer cells. Briefly, after prehybridization at 55°C for 2 h, frozen sections and cell climbing piece were hybridized with specific Cy3-labeled circFAM169A probes at 37°C overnight, and dyed with DAPI. Slides were photographed with a fluorescence microscope (Nikon, Japan).

### Statistical analysis

The experimental data were analyzed by GraphPad Prism 7. Survival rates were analyzed by using Kaplan Meier survival analysis. Student’s t-test was used for comparison of two groups, and one-way ANOVA was used for comparison of multiple groups, and p value less than 0.05 were considered to be statistically significant.

## Results

### RPS6KA2 was lowly expressed and played a role as a tumor suppressor in ovarian cancer

**Figure 1:**
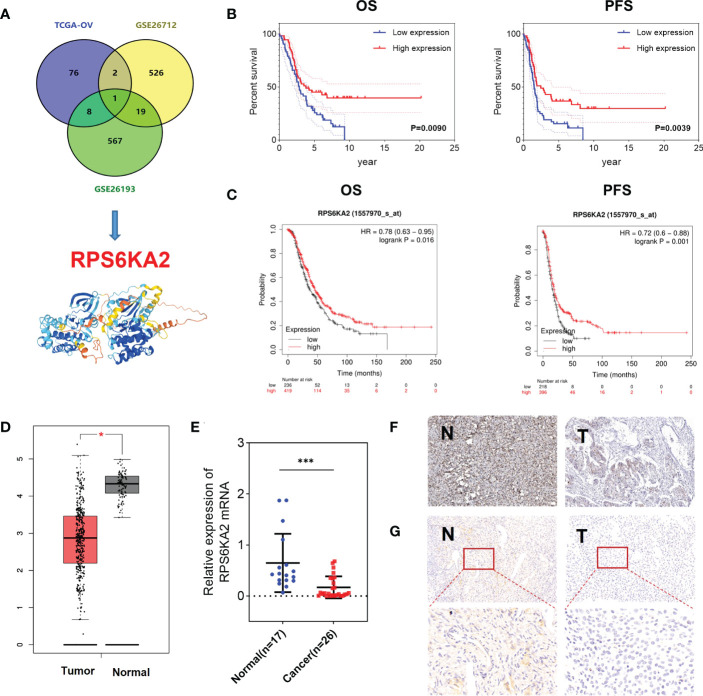
RPS6KA2 was lowly expressed and played a role as a tumor suppressor in ovarian cancer. **(A)** Data-mining analysis of ovarian cancer prognosis related-genes in TAGA, GSE26712 and GSE26193. **(B, C)** Subsistence analysis of RPS6KA2 in ovarian cancer patients with different datasets. **(D)** TCGA data of RPS6KA2 expression levels in ovarian cancer were analyzed. **(E)** The mRNA levels of RPS6KA2 in tumor tissues and normal tissues from our hospital. **(F)** The representative photographs of RPS6KA2 IHC staining in tumor tissues and normal tissues from the Human Protein Atlas database. **(G)** The representative photographs of RPS6KA2 IHC staining in tumor tissues and normal tissues from our ovarian cancer specimen. (* indicates p<0.05, *** indicates p<0.001).

In our study, 87 prognosis-related genes from TCGA-OV, 548 prognosis-related genes from GSE26712 dataset and 595 prognosis-related genes from GSE26193 dataset were identified. RPS6KA2 was the unique prognosis-related gene present in all three datasets (**Figure 1A**), indicating that RPS6KA2 may play an important role in the development and prognosis of ovarian cancer. Kaplan–Meier plotter was used to evaluate the overall survival (OS) and progression-free survival (PFS) of RPS6KA2 using GSE26193 (**Figure 1B**) and TCGA clinical data (**Figure 1C**). The high expression of RPS6KA2 had a better OS and PFS. Subsequently, we verified the expression of RPS6KA2 at the mRNA level by TCGA (**Figure 1D**) and the specimens from our hospital. We analyzed its mRNA degree of expression in 26 tumor tissues and 17 normal tissues by qRT-PCR, we found that RPS6KA2 mRNA levels were significantly lower in ovarian cancer patient tissues compared to those in normal tissues (**Figure 1E**). Moreover, we analyzed the difference in protein expression of RPS6KA2 in tumor and normal tissues using immunohistochemistry (IHC) in the Human Protein Atlas database (**Figure 1F**) and our ovarian cancer specimens (**Figure 1G**). Histochemistry score (H-score) in normal tissues (N) were 13.5 and 3.8 times higher than those in tumor tissues (T), which confirmed a low protein expression of RPS6KA2 in ovarian cancer, especially in cytoplasm. Additionally, we analyzed the association between RPS6KA2 expression levels and clinicopathological characteristics from ovarian cancer patients. The expression level of RPS6KA2 was significantly correlated with the diameter and ascites (**Table 1**). These results showed that RPS6KA2 was lowly expressed and may play a role as a tumor suppressor in the development of ovarian cancer.

**Table 1:** Comparison of clinicopathological profiles of ovarian cancer patients between the low and high RPS6KA2 expression groups.

Characteristic		RP56KA2 expression		Chi-Square	P value
		Low (n=24)	High (n=26)		
Age (year)	≥ 50	16	19	0.244	0.621
	< 50	8	7		
Diameter (cm)	≥ 8	19	14	8.099	0.004
	< 8	5	12		
FIGO stage	I-II	12	12	0.073	0.785
	III-IV	12	14		
Lymphatic	Absent	13	10	1.239	0.265
metastasis	Present	11	16		
CA125 (U/ml)	≥ 500	9	9	0.451	0.832
	< 500	15	17		
Histological grade	G1-G2	11	12	0.001	0.982
	G3	13	14		
Histological type	Serous	18	22	0.721	0.396
	Others	6	4		
Ascites	Absent	8	20	9.623	0.002
	Present	16	6		

### RPS6KA2 inhibited the proliferation of ovarian cancer cells

**Figure 2:**
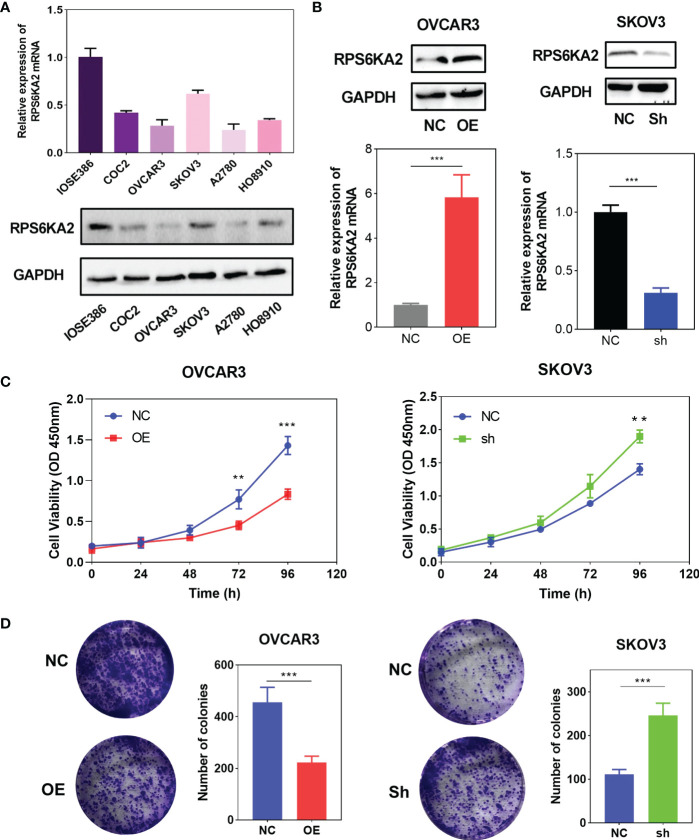
RPS6KA2 inhibited the proliferation of ovarian cancer cells. **(A)** The mRNA and protein expression of RPS6KA2 in the IOSE386 and ovarian cancer cell lines including HO8910, SKOV3, OVCAR3, A2780 and COC2. **(B)** The mRNA and protein expression levels of RPS6KA2 were examined in OVCAR3 cells transfected with OE and SKOV3 cells transfected with Sh. **(C)**The proliferation of RPS6KA2 overexpressed and knockdowned ovarian cancer cells was measured by CCK-8 assay. **(D)** The colony formation was assessed in ovarian cancer cells and statistical significance was analyzed based on the numbers of colonies.(** indicates p<0.01, *** indicates p<0.001).

**Figure 3:**
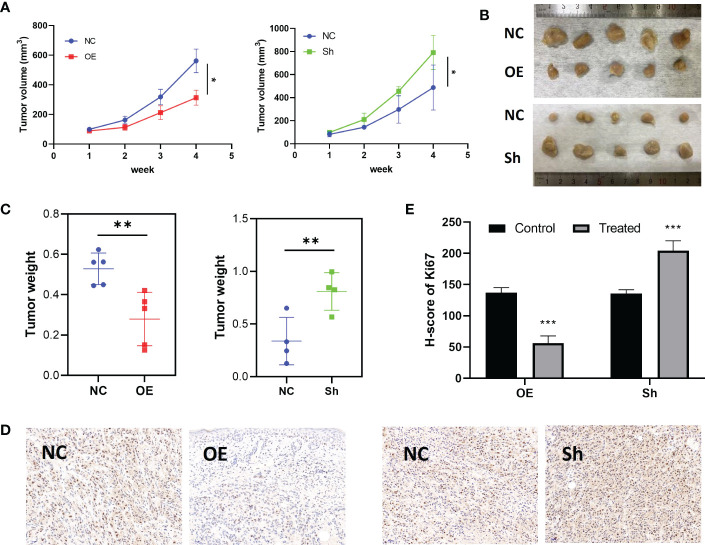
RPS6KA2 inhibited the growth of ovarian cancer *in vivo*. **(A)** Tumor growth curves in each group. **(B)** Pictures of tumors which were formed in nude mice. **(C)** Tumor weights in each group. **(D)** Ki67 staining was detected in ovarian cancer subcutaneous xenograft model by IHC. **(E)** H-score of Ki67 staining. (* indicates p<0.05, ** indicates p<0.01, *** indicates p<0.001).

To explore the inhibition effect of RPS6KA2 on ovarian cancer, we tested the mRNA and protein expression of RPS6KA2 in five ovarian cancer cell lines (HO8910, SKOV3, OVCAR3, A2780 and COC2) and a normal ovarian cell line (IOSE386), identical with the tumor tissue profiles, ovarian cancer cells expressed lower levels of RPS6KA2 than that in normal cell (**Figure 2A**). Based on the expression of RPS6KA2, we selected OVCAR3 and SKOV3 for follow-up studies. Firstly, The OVCAR3 was transfected with negative control (NC) and overexpressed RPS6KA2 (OE), and the SKOV3 was transfected with scramble control (NC) and knockdown RPS6KA2 (Sh). The transfection efficiency of RPS6KA2 lentivirus was verified by WB and PCR, and the results showed that RPS6KA2 expression could be effectively manipulated in ovarian cancer cells (**Figure 2B**). To further explore whether RPS6KA2 inhibits the proliferation of ovarian cancer cells, we conducted CCK-8 and colony formation assays. Compared with control cells, the OE group of OVCAR3 cells decreased the cell viability and attenuated the colony formation capability of ovarian cancer cells. In contrast, SKOV3 cells transfected with RPS6KA2 shRNA enhanced the proliferation and colony formation capability of ovarian cancer cells (**Figure 2C, D**). The subcutaneous tumor formation assay was performed in nude mice to determine the influence of RPS6KA2 on ovarian cancer proliferation *in vivo*. We found that the tumor volume and weight were inhibited with the overexpression of RPS6KA2 in OVCAR3 group and promoted with the knockdown of RPS6KA2 in SKOV3 group (**Figures 3A–C**). H-score of Ki-67 staining showed that the proliferative potential was lower in OE group than corresponding NC group while higher in Sh group than corresponding NC group (**Figure 3D, E**). These results fully indicated that RPS6KA2 could inhibit the growth and proliferation of ovarian cancer cells.

### RPS6KA2 inhibited development of ovarian cancer cell *via* the MAKP pathway

**Figure 4:**
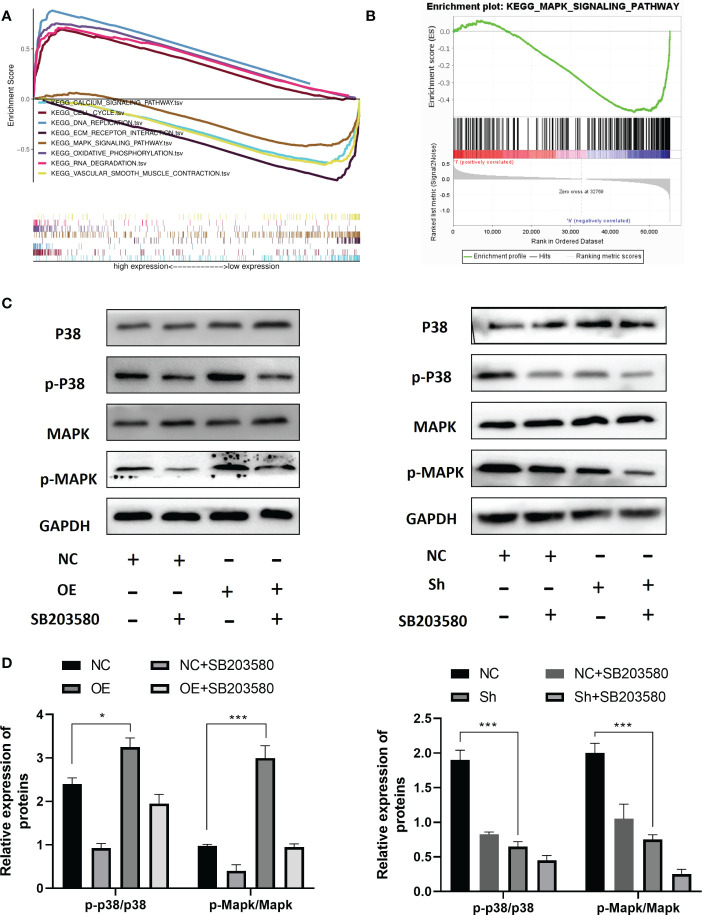
RPS6KA2 inhibited development of ovarian cancer cell *via* the MAKP pathway. **(A, B)** GSEA analysis showed the relevance between MAKP signaling pathway and RPS6KA2. **(C, D)** The protein expression levels of p-p38/p38 and MAPK/p-MAPK in OVCAR3 and SKOV3 cells. (* indicates p<0.05, *** indicates p<0.001).

To investigate the molecular mechanism of RPS6KA2 in ovarian cancer, we conducted gene set enrichment analysis (GSEA) according to the expression level of RPS6KA2. Top 8 KEGG pathways are CALCIUM SIGNALING PATHWAY, CELL CYCLE, DNA REPLICATION, ECM-RECEPTOR INTERACTION, MAPK SIGNALING PATHWAY, OXIDATIVE PHOSPHORYLATION, RNA DEGRADATION and VASCULAR SMOOTH MUSCLE CONTRACTION (**Figure 4A**). Among these pathways, The MAPK pathway which was usually associated with tumor development was significantly enriched (**Figure 4B**). Then we detected the p-p38/p38 and MAPK/p-MAPK protein in OVCAR3 cells transfected with NC and OE and SKOV3 cells transfected with NC and Sh. The RPS6KA2 overexpression increased the expressions of p-p38 and p-MAPK while the RPS6KA2 knockdown decreased the expressions of p-p38 and p-MAPK (**Figure 4C, D**). It suggested that the MAKP signaling pathway may be involved in RPS6KA2-mediated ovarian cancer progression. To further demonstrate the role of MAKP signaling pathway in the RPS6KA2 regulating ovarian cancer cells proliferation. MAKP inhibitor (SB203580) was added into OE-OVCAR3 cells and Sh-SKOV3 cells. We found that SB203580 and RPS6KA2-Sh synergistically inhibited p38/MAPK phosphorylation in ovarian cancer cells, while SB203580 partially rescued p38/MAPK activation caused by RPS6KA2-OE. These data illustrated that RPS6KA2 regulated ovarian cancer proliferation by activating the p38/MAKP signaling pathway.

### miR-19a-3p, miR-106a-5p and miR-519d-3p targeted RPS6KA2

**Figure 5:**
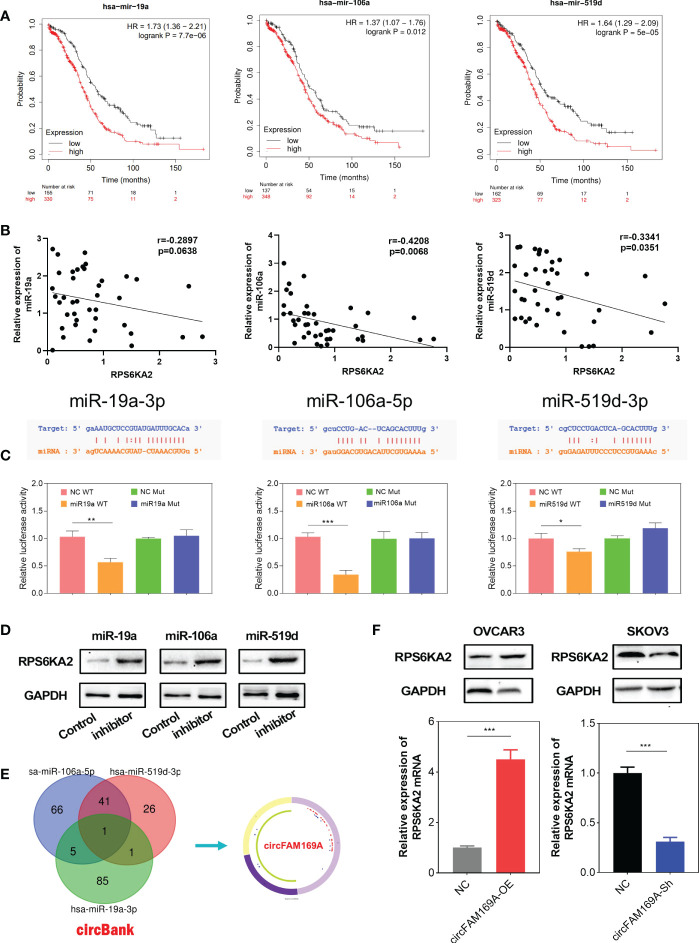
miR-19a-3p, miR-106a-5p and miR-519d-3p targeted RPS6KA2. **(A)** Prognostic estimation of miR-19a-3p, miR-106a-5p and miR-519d-3p in ovarian cancer. **(B)** The correlation between miRNAs and RPS6KA2 expression. **(C)** Dual-Luciferase reporter assay was performed to verify the location between RPS6KA2 and miR-19a-3p, miR-106a-5p and miR-519d-3p. **(D)** The expression of RPS6KA2 after inhibiting upstream miRNAs. **(E)** circFAM169A was screened through overlapping websites of circBank. **(F)** The mRNA and protein level of RPS6KA2 after transfection with circFAM169A mimics and circFAM169A inhibitor. (* indicates p<0.05, ** indicates p<0.01, *** indicates p<0.001).

To further explore molecular mechanism of RPS6KA2, we used miRBase to predict all the related miRNAs which target RPS6KA2. Thereinto, miR-19a-3p, miR-106a-5p and miR-519d-3p were filtered as the prognosis related targets. **Figure 5A** showed the prognostic relevance between these miRNAs and ovarian cancer. Moreover, miR-19a-3p, miR-106a-5p and miR-519d-3p were negatively correlated with RPS6KA2 expression in ovarian cancer tissues(**Figure 5B**). Further, Dual-Luciferase reporter assay showed that miR-19a-3p, miR-106a-5p and miR-519d-3p reduced the activity of the luciferase reporter fused to RPS6KA2, but did not fuse to the Mut version (**Figure 5C**). In addition, the expression of RPS6KA2 was increased by inhibiting upstream of miRNAs, further illustrating the correlation between these miRNAs and RPS6KA2 (**Figure 5D**). Afterwards, circBank database (http://www.circbank.cn) was screened to predict the ceRNA of miR-19a-3p, miR-106a-5p and miR-519d-3p. Then, circFAM169A was identified (**Figure 5E**). We transfected circFAM169A overexpression plasmid in OVCAR3 cells and circFAM169A shRNA in SKOV3 cells. The mRNA and protein level of RPS6KA2 was increased when circFAM169A mimicked in OVCAR3 cells and decreased when circFAM169A inhibited in SKOV3 cells (**Figure 5F**).

### circFAM169A can sponge miR-106a-5p and miR-519d-3p

**Figure 6:**
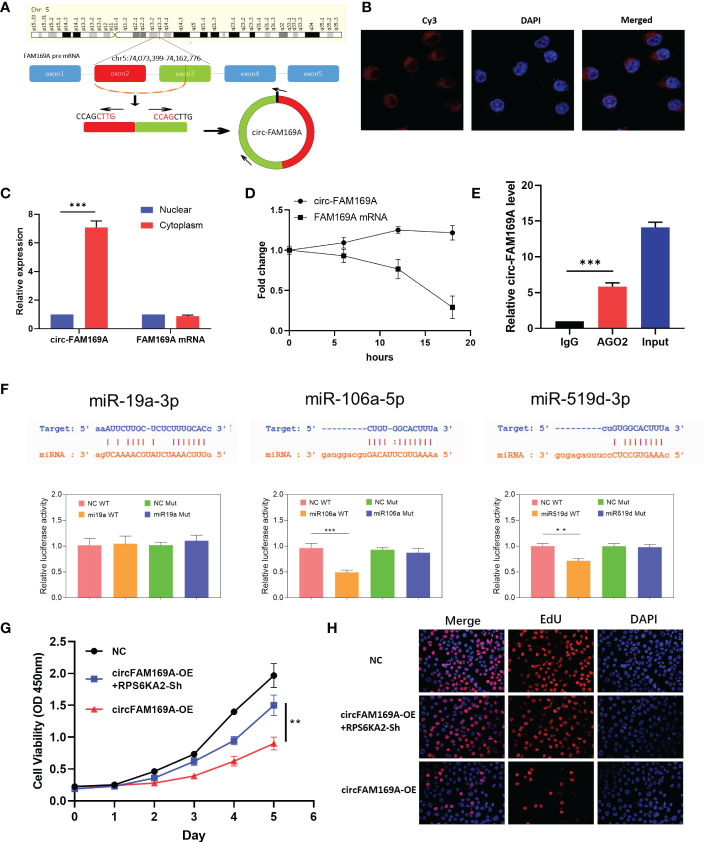
circFAM169A was the sponge of miR-106a-5p and miR-519d-3p. **(A)** The genomic loci of circFAM169A in FAM169A mRNA. **(B, C)** The location of circFAM169A was detected by FISH assay and nucleoplasmic separation assay. **(D)** qRT-PCR of circFAM169A and FAM169A mRNA during differentiation. **(E)** Enrichment of circFAM169A by AGO2 antibody and IgG was assessed by RIP assay. **(F)** Dual-Luciferase reporter assays were performed to verify the predicted sites between circFAM169A and miRNAs. **(G, H)** Cell proliferative ability was assessed by CCK-8 assay and EdU assay. (** indicates p<0.01, *** indicates p<0.001).

circFAM169A was derived from chr5:74,073,399-74,162,776 of FAM169A preRNA (**Figure 6A**). Considering the connection between circFAM169A and miR-19a-3p, miR-106a-5p and miR-519d-3p, we assumed that circFAM169A acts as a sponge for miR-19a-3p, miR-106a-5p and miR-519d-3p. By FISH and nucleoplasmic separation assay, circFAM169A was abundant in the cytoplasm (**Figure 6B, C**). Actinomycin D assay were used to validate the stability of circFAM169A, one of the peculiarities of circRNAs and the results showed that FAM169A mRNA was gradually degraded as time gone by, while circFAM169A remained stable (**Figure 6D**). As miRNAs function mainly in an RNA–induced silencing complex (RISC) dependent manner, which requires the participant of a key protein, Argonaute 2 (AGO2). Therefore, it’s essential to examine whether circFAM169A can bind to AGO2. RNA immunoprecipitation (RIP) assays were performed and the results showed that circFAM169A was efficiently pulled down by AGO2 antibody (**Figure 6E**). At last, Dual-Luciferase reporter assay showed that miR-106a-5p and miR-519d-3p reduced the activity of the luciferase reporter fused to wild-type circFAM169A (**Figure 6F**). Further studies confirmed that overexpression of circFAM169A can also inhibit the proliferation and cell viability of ovarian cancer through CCK-8 and EdU assay, and inhibition of RPS6KA2 can partially counteract the effects of circFAM169A (**Figures 6G, H**).

## Discussion

The RPS6K family are highly conserved serine-threonine kinases (32, 33). The functions of RPS6K family has been proposed, which include phosphorylation of c-Fos, oestrogen receptor, nuclear factor-kappa B/IkBa, CREB, histones, Myt1, glycogen synthase kinase-3, BAD and regulation of gene expression (34). RPS6KA2 was first isolated in 1994 and encoded a protein of approximately 90 kDa that acts downstream of MAP kinases to transduce a variety of signals from the cell surface induced by growth factors or stress to the nucleus (35, 36).RPS6KA2 has been confirmed to play an important role in prostate cancer, pancreatic cancer, colon or rectal cancer (37–39). In our study, we found RPS6KA2 was related to the prognosis of ovarian cancer through TCGA, GSE26712 and GSE26193, the highly expression of RPS6KA2 had a better prognosis in ovarian cancer. This was supported by further studies, in which we found that RPS6KA2 was downregulated in ovarian cancer tissues and cell lines and promoted the proliferative capacity of ovarian cancer cells. GSEA showed RPS6KA2 inhibited cell proliferation and promoted cell apoptosis in ovarian cancer through MAPK signaling pathway. Moreover, low expression of RPS6KA2 increased the protein of p-p38 and p-MARK. These findings may provide new opinions for the development of tumor targeted agents.

circRNAs are abundant in the transcriptome of eukaryotic cells, which are closed circular RNAs formed by covalent bonds (40, 41). Most circRNAs are conserved in species. They are resistant to RNases, and often exhibit tissue-specific or developmental stage-specific expression (42). More and more researches conformed that circRNAs play key regulatory roles in cancer progression, different circRNA play different functions (43, 44). The high expression of circRNA_100876 had the significantly shorter overall survival time in non-small cell lung cancer patients (45). And circRNA_102,958 was significantly overexpressed in gastric cancer and it may be a potential novel biomarker for the diagnosis of gastric cancer (46). Moreover, circRNA-RAPG EF5 promoted papillary thyroid cancer cell proliferation, invasion, and migration (47). We found that miR-19a-3p, miR-106a-5p and miR-519d-3p were as the targets of RPS6KA2. Bioinformatics analysis showed the prognosis correlation between ovarian cancer patients and these miRNAs. Then we identified circFAM169A as the common target circRNA of miR-19a-3p, miR-106a-5p and miR-519d-3p. What’s more, the expression of RPS6KA2 was positively correlated with circFAM169A in ovarian cancer cells. Further studies revealed that circFAM169A resides mainly in the cytoplasm, not the nucleus, and has high stability. Dual-Luciferase reporter assay verified that circFAM169A facilitated RPS6KA2 expression by restraining miR-106a-5p and miR-519d-3p. Thus, circFAM169A may be able to work as a new target for ovarian cancer treatment.

## Conclusions

To sum up, we confirmed the significant role of RPS6KA2 in ovarian cancer. Our data demonstrated that circFAM169A functioned as the ceRNA for miR-106a-5p and miR-519d-3p to facilitate RPS6KA2 expression and inhibit ovarian cancer cell proliferation through p38/MAPK signaling pathway. Our study enriches the research of the molecular biological mechanism of ovarian cancer from the circRNA-miRNA-mRNA network perspective, and may provide a potential treatment approach for ovarian cancer patients.

## Data availability statement

The original contributions presented in the study are included in the article/supplementary material. Further inquiries can be directed to the corresponding author.

## Ethics statement

The studies involving human participants were reviewed and approved by Zhejiang Provincial People’s Hospital. The patients/participants provided their written informed consent to participate in this study. The animal study was reviewed and approved by Zhejiang Provincial People’s Hospital.

## Author contributions

Study conception and design: ZF and CL. Study conduct: ZF, CD, WG. Data analysis: ZF and CD. Manuscript preparation: ZF and CL. All authors contributed to the article and approved the submitted version.
